# Changes in Branched-Chain Amino Acids One Year after Sleeve Gastrectomy in Youth with Obesity and Their Association with Changes in Insulin Resistance

**DOI:** 10.3390/nu15173801

**Published:** 2023-08-30

**Authors:** Imen Becetti, Meghan Lauze, Hang Lee, Miriam A. Bredella, Madhusmita Misra, Vibha Singhal

**Affiliations:** 1 Division of Pediatric Endocrinology, Mass General for Children and Harvard Medical School, Boston, MA 02114, USA; mmisra@mgh.harvard.edu (M.M.); vsinghal1@mgh.harvard.edu (V.S.); 2 Neuroendocrine Unit, Massachusetts General Hospital and Harvard Medical School, Boston, MA 02114, USA; 3 Biostatistics Center, Massachusetts General Hospital, Boston, MA 02114, USA; 4 Department of Radiology, Musculoskeletal Imaging and Interventions, Massachusetts General Hospital, Harvard Medical School, Boston, MA 02114, USA; 5 Pediatric Program, MGH Weight Center, Massachusetts General Hospital, Boston, MA 02114, USA

**Keywords:** branched-chain amino acids, isoleucine, leucine, valine, obesity, sleeve gastrectomy, youth, insulin resistance

## Abstract

Adults with obesity have a reduction in branched-chain amino acid (BCAA) levels following metabolic and bariatric surgery (MBS), which is hypothesized to contribute to the metabolic advantages of MBS. We examined this relationship in 62 youth 13–24 years old with severe obesity (47 female) over 12 months. Thirty had sleeve gastrectomy (SG) and 32 were non-surgical controls (NS). We measured fasting insulin, glucose, glycated hemoglobin (HbA1c), isoleucine, leucine, and valine concentrations, and post-prandial insulin and glucose, following a mixed meal tolerance test. Twenty-four-hour food recalls were collected. At baseline, groups did not differ in the intake or the serum levels of BCAAs, HbA1C, HOMA-IR, Matsuda index, insulinogenic index, or oral Disposition index (oDI). Over 12 months, SG vs. NS had greater reductions in serum BCAAs, and SG had significant reductions in BCAA intake. SG vs. NS had greater reductions in HbA1c and HOMA-IR, with increases in the Matsuda index and oDI. In SG, baseline leucine and total BCAA concentrations were negatively correlated with the baseline Matsuda index. Reductions in serum leucine were positively associated with the reductions in HOMA-IR over 12 months. These associations suggest a potential role of BCAA in regulating metabolic health. Reducing dietary intake and serum BCAA concentrations may reduce insulin resistance.

## 1. Introduction

The prevalence of obesity has dramatically increased, with most recent estimates indicating that 19.3% of youth had obesity in 2017–2018 (with subjects having a body mass index (BMI) at or above the 95th percentile for age and sex meeting the definition of obesity) [1]. The rise in obesity rates in youth has been accompanied by a surge in obesity-related metabolic comorbidities, including type 2 diabetes mellitus (T2DM), dyslipidemia nonalcoholic fatty liver disease (NAFLD), hypertension, and obstructive sleep apnea [2,3]. Further, development of T2DM in adolescents and young adults carries an increased risk of morbidity and mortality compared to adult-onset T2DM, with faster deterioration of beta cell function (BCF) and a higher rate of complications such as diabetic kidney disease, cardiovascular disease, and peripheral neuropathy [4,5,6].

Metabolic and bariatric surgery (MBS), including sleeve gastrectomy (SG) and Roux-en-Y gastric bypass (RYGB), has been increasingly performed in youth in the last decade with the rising obesity rates [7]. Studies have shown that adolescents and young adults undergoing MBS experience marked weight reduction and improved metabolic health, including remission of T2DM [8,9,10].

Recently, there has been growing evidence highlighting the association between branched-chain amino acids (BCAAs—isoleucine, leucine, and valine) and certain obesity-related comorbidities, particularly insulin resistance (IR) and T2DM. Studies have demonstrated higher BCAA levels in individuals with obesity and T2DM [11,12,13]. Further, higher BCAA levels are strongly correlated with measures of insulin resistance in individuals with obesity and/or T2DM [11,12,14,15], and are predictors of future risk of T2DM across the age spectrum [16,17,18,19]. Changes in BCAA concentrations are also associated with reduced insulin resistance following weight loss via lifestyle interventions [20]. These findings support the hypothesis that BCAAs may play a significant role in the pathogenesis of IR and T2DM [21,22,23,24,25,26].

Studies in adults have demonstrated a reduction in BCAA concentrations following SG or RYGB [27,28,29,30,31], which are associated with reductions in insulin resistance [32]. To our knowledge, these findings have not yet been investigated in youth. Adolescence is characterized by a physiological increase in insulin resistance, which is further exacerbated by obesity and eating patterns during adolescence [33]. Thus, data from adults may not be completely translatable to adolescents. This study aimed to examine, for the first time, changes in BCAA concentrations 12 months following SG, and associations between BCAA levels and markers of IR and BCF before and after surgery. Our hypothesis was that BCAA levels would decrease after SG and that higher BCAA concentrations would be associated with increased IR.

## 2. Materials and Methods

### 2.1. Participant Selection

We enrolled 62 adolescents and young adults, 13–24 years of age, with severe obesity (defined as BMI of ≥35 kg/m^2^ or ≥120% of the 95th percentile BMI for sex and age with the presence of at least one obesity-related complication, or a BMI ≥ 40 kg/m^2^ or ≥140% of the 95th percentile BMI, to meet the requirements for MBS). Thirty participants (23 female and 7 male) underwent SG, a decision made by the treating physician, participant, and their family. Thirty-two participants (24 female and 8 male) were non-surgical (NS) controls who received routine lifestyle intervention. Recruitment was performed from tertiary care obesity treatment centers providing lifestyle and surgical weight management interventions. Exclusion criteria were designed according to the primary endpoint of this study, which focused on bone endpoints. Thus, participants with a history of bone disorders, medications that may have an effect on bone metabolism (exceptions include vitamin D, calcium, and hormonal contraceptives), untreated thyroid disease, substance abuse per DSM-5 criteria, smoking ≥ 10 cigarettes/day, pregnancy, or breastfeeding were excluded. This study was conducted at the Translation and Clinical Research Center of Massachusetts General Hospital (MGH). This study received approval from the Partners/Mass General Brigham Institutional Review Board and remained in compliance with the Health Insurance Portability and Accountability Act. We obtained written informed consent from participants ≥ 18 years and parents of participants < 18 years, and informed assent from participants 13–17 years old.

### 2.2. Study Visits

To ensure eligibility, participants initially completed a screening visit. Participants in the SG group were examined at baseline (within a month prior to surgery) and 12 months after SG. Study visits were also performed at baseline and 12 months for NS control subjects, who were counseled on standard diet and exercise. Participants presented to the TCRC after an overnight fast. Visits included a medical history, physical examination, 24 h food recall, and anthropometric measurements. Weight was measured using an electronic scale to the nearest 0.1 kg. Height was calculated as the mean of three measurements using a wall-mounted stadiometer. BMI was calculated as weight in kg/(height in meters)^2^. Participants had fasting blood tests for insulin, glucose, glycated hemoglobin (HbA1c), isoleucine, leucine, and valine. Following a mixed meal tolerance test (MMTT) where participants consumed 360 mL of Boost; post-prandial insulin and glucose levels were also measured at 30, 60, 90, and 120 min. Due to manufacturing changes over the course of this study, Boost formulations differed in dextrose content (50 vs. 75 g) but were otherwise identical. Thus, we controlled for Boost formulation in our analyses. Not all participants had all data available, but at least 18 surgical and 19 non-surgical participants had complete data.

### 2.3. Calculation of IR and BCF Markers

Homeostatic Model Assessment for Insulin Resistance (HOMA-IR), a marker of IR, was calculated as the following: (fasting insulin, uIU/mL) × (fasting glucose, mg/dL)/405 [34]. The insulinogenic index, a marker of insulin secretion and sensitivity, was calculated as the ratio of change in insulin to change in glucose from 0 to 30 min (ΔI30/ΔG30) [35]. The oral disposition index (oDI) was calculated as the insulinogenic index (ΔI30/ΔG30) multiplied by 1/fasting insulin level and is a measure of BCF adjusted for insulin sensitivity [36,37]. Matsuda index, which represents insulin sensitivity in hepatic and peripheral tissue [38], was calculated using the published formula: (10,000/square root of [fasting glucose × fasting insulin] × [mean glucose × mean insulin during MMTT]) [39].

### 2.4. Twenty-four Hour Food Recall

A trained registered dietician completed the 24 h food recall and recorded all foods and beverages consumed over the 24 h preceding the baseline or 12-month visit. The Nutrition Data System for Research (NDSR), which is a computer-based dietary analysis program, was used to analyze the data.

### 2.5. Biochemical Analysis

Serum insulin concentrations were measured by an ultrasensitive chemiluminescence immunoassay (Beckman Coulter, Fullerton, CA, USA; intra-assay coefficient of variation (CV) 2.0–4.2%, interassay CV 3.1–5.6%, sensitivity 0.03 uIU/mL). Serum glucose levels were measured by a glucose hexokinase assay (Roche Diagnostics, Indianapolis, IN, USA; intermediate precision (day-to-day) CV of 1.1–1.2%, sensitivity 2 mg/dL). Measurements of HbA1c levels were performed using boronate affinity high performance liquid chromatography (Trinity Biotech, Jamestown, NY, USA; intra- and interassay CV < 2%, sensitivity 3.8%). Serum isoleucine, leucine, and valine were measured by liquid chromatography—tandem mass spectroscopy. Results were standardized by using a ratio of the sample value to the value of the nearest pooled reference (derived from a pool of the 20 nearest samples), multiplied by the median of all reference values for each amino acid [40]. Total BCAA levels were calculated as the sum of the individual BCAA levels.

### 2.6. Statistical Analysis

Statistical analyses were completed using JMP Statistical Discovery Software (Version 16, SAS Institute, Carey, NC, USA). We present continuous data as mean ± standard error of the mean (SEM) or median (interquartile range), and categorical data as absolute numbers.

We compared baseline characteristics and 12-month changes in SG vs. NS groups using Student’s *t*-test or the Wilcoxon Rank Sum test according to the data distribution. We performed within group comparisons using paired t-tests or Wilcoxon sign rank tests according to data distribution. Linear regression analyses were performed to determine associations between serum levels of BCAAs and markers of IR and BCF. Spearman’s correlation coefficients are reported. General linear models (multivariate regression analysis) were used to determine differences between groups after controlling for possible covariates, such as type of Boost drink consumed (categorical covariate) or BMI (continuous covariate). *p* ≤ 0.05 was used to denote significance.

## 3. Results

### 3.1. Baseline Characteristics

SG and NS groups did not differ for baseline age, sex, race, or BMI z-score, except for weight and BMI, which were higher in the SG compared to the NS group (Table 1). The groups did not differ at baseline for dietary intake or serum levels of isoleucine, leucine, valine, or total BCAAs (Table 1). There were also no baseline differences in HOMA-IR, HbA1c, insulinogenic index, Matsuda index, or oDI between groups (Table 1).

### 3.2. Changes in Weight and BMI

Over 12 months, the SG group demonstrated significant decreases in weight, BMI, and BMI z-score compared to the NS group (*p* < 0.0001, Table 1). The SG group showed within-group reductions in weight, BMI, and BMI z-score, while the NS group had within-group reduction in the BMI z-score (Table 1).

### 3.3. Changes in Dietary Intake and Serum Levels of BCAAs

**Figure 1:**
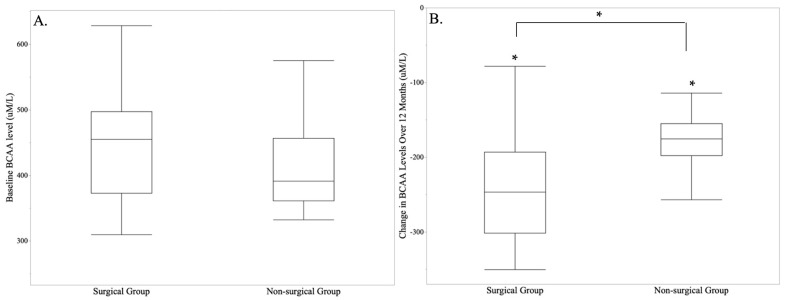
Serum total BCAA levels at baseline (**A**) and changes over 12 months (**B**) in the surgical and non-surgical groups. Total BCAA levels decreased significantly in the surgical group over 12 months. * *p* < 0.05.

Changes in dietary intake of isoleucine, leucine, valine, or total BCAAs did not differ significantly between SG and NS groups (Table 1). However, the SG group did have significant within-group reductions in dietary intake of individual and total BCAAs (Table 1). Serum levels of isoleucine, leucine, valine, and total BCAAs decreased significantly in SG compared to NS groups (*p* = 0.02, *p* = 0. 0001, *p* < 0.0001, and *p* = 0.0004, respectively) with significant within-group reductions following SG (Table 1). There was also a within-group decrease in total BCAA serum levels in the NS group (Table 1). Figure 1 demonstrates baseline and changes in serum total BCAA levels in both groups.

### 3.4. Changes in IR and BCF Markers

The SG group compared to the NS group demonstrated greater decreases in HOMA-IR (*p* < 0.0001) and HbA1c (*p* = 0.002), as well as within-group decreases in these parameters over 12 months following SG (Table 1). There was also a significant within-group reduction in HbA1c in the NS group (Table 1). Matsuda index increased significantly in SG compared to NS groups (*p* < 0.0001), which persisted after controlling for the type of Boost drink consumed during MMTT (Table 1). Additionally, there were significant within-group increases in the Matsuda index in the surgical group, along with significant within-group reductions in the NS group (Table 1). oDI also increased significantly in SG compared to NS groups (*p* = 0.02), with significant within-group increases in the surgical group (Table 1); however, this difference was lost after controlling for the type of Boost drink consumed during MMTT. The insulinogenic index did not show significant within or between group changes (Table 1).

### 3.5. Correlations between Serum BCAA Levels and Weight

Baseline serum valine level was positively associated with baseline weight in the whole group (ρ = 0.36, *p*-value = 0.02). Other BCAAs were not associated with baseline weight. Changes in individual and total BCAA levels over 12 months were positively associated with weight or BMI changes over 12 months (*p*-value ≤ 0.005); however, these did not persist after controlling for group status.

### 3.6. Correlations between Baseline Serum BCAA Levels and the Markers of IR and BCF

In the SG group, baseline serum leucine level was positively correlated with baseline HOMA-IR (ρ = 0.53, *p*-value = 0.02), which did not persist after controlling for baseline BMI, and negatively correlated with baseline Matsuda index (ρ = −0.61, *p*-value = 0.01). Total BCAA level at baseline was also negatively correlated with baseline Matsuda index (ρ = −0.54, *p*-value = 0.03). Associations with baseline Matsuda index persisted after controlling for baseline BMI and type of Boost drink consumed during MMTT. No associations were detected between other BCAA levels and the markers of IR or BCF at baseline.

### 3.7. Correlations between Changes in Serum BCAA Levels and Changes in the Markers of IR and BCF

The reduction in serum levels of leucine over 12 months was positively correlated with reductions in HOMA-IR over 12 months in the whole cohort (ρ = 0.53, *p*-value = 0.0007), and this association remained significant after controlling for the change in BMI. No associations were detected between other BCAA levels and the markers of IR and BCF over 12 months, or within the SG group.

## 4. Discussion

Youth with severe obesity sustained the expected reductions in weight, BMI, and BMI z-score 12 months following SG. Further, they demonstrated significant reductions in the serum levels of isoleucine, leucine, valine, and total BCAAs 12 months following SG. Our data also show significant decreases in HOMA-IR and HbA1c, with significant increases in Matsuda index and oDI, reflecting improved insulin sensitivity and BCF over the 12-month study period after SG (as expected). At baseline, serum valine level was positively associated with weight. Additionally, there was a positive correlation between baseline serum leucine level and HOMA-IR at baseline; however, this association was primarily driven by baseline BMI. Moreover, baseline serum leucine and total BCAA levels were negatively correlated with Matsuda index at baseline. Over 12 months, reductions in serum leucine levels were positively associated with reductions in HOMA-IR. To the best of our knowledge, this is the first study in youth that shows changes in serum BCAA levels after SG, and their associations with the markers of IR and BCF.

BCAA levels decreased significantly over 12 months following SG in youth, consistent with our hypothesis and prior studies in adults undergoing SG or RYGB [27,28,29,30,31]. A recent study demonstrated decreases in serum BCAA concentrations following MBS that were not observed in patients receiving liraglutide; however, these reductions were lost after adjusting for weight [41]. The mechanism by which this occurs has been under debate. Some studies have demonstrated that genes involved in BCAA metabolism are downregulated in human and animal models with obesity [42,43,44,45,46], with an upregulation of these genes following MBS [42,43]. Thus, these studies suggest that BCAA concentrations are elevated in obesity secondary to slower metabolism, which increases following MBS, and thereby results in a reduction in serum BCAA levels. Conversely, a more recent study showed that increased BCAA metabolism in the setting of obesity contributes to higher BCAA concentrations, which decrease following SG due to reduced protein breakdown [27]. In our cohort, we detected a positive association between baseline valine level and baseline weight. Additional studies investigating associations between BCAA concentrations, body habitus, and weight loss interventions are necessary to clarify the potential causal relationships.

Data from our cohort show a positive correlation between serum leucine and HOMA-IR at baseline that was primarily driven by baseline BMI, whereas baseline serum leucine and total BCAA levels were negatively correlated with Matsuda index, in which a lower level is indicative of higher IR [39]. These associations were observed at baseline in the group undergoing SG, but not in the group receiving standard therapy, likely because the baseline weight and BMI in the SG was significantly higher than in the NS group. Further, there was a positive correlation between the 12-month changes in serum leucine and HOMA-IR. Our findings indicate that the reduction of serum BCAA concentrations following SG is associated with improved insulin sensitivity, and consistent with prior studies demonstrating a correlation between higher BCAA concentrations and IR in individuals with obesity [11,12,14]. We did not observe, however, associations between BCAA concentrations and markers of BCF (insulinogenic index or oDI) in our cohort.

As discussed above, the underlying mechanisms resulting in increased serum BCAA levels in the setting of obesity remain under investigation. Similarly, while some studies demonstrate that serum BCAAs have a direct role in the development of IR by acting as signaling molecules [11,25,47,48,49,50,51], other reports alternatively suggest that higher serum BCAA concentrations are a consequence of IR [52,53]. Further, whether a reduction of serum BCAAs following SG mediates IR reduction remains of debate, as the positive effects of SG on body weight and insulin sensitivity were still observed in a mouse model with elevated serum BCAA concentrations due to impaired BCAA metabolism or increased dietary intake of BCAAs [32]. Our data suggest that changes in the levels of leucine are independently associated with changes in IR after controlling for changes in BMI, suggesting that BCAA may have a direct impact on IR. Future studies dissecting the causal relationship between BCAAs and IR are needed to answer these questions and determine whether interventions targeted at decreasing serum BCAA levels may have a therapeutic role in the treatment of obesity, IR, and type 2 diabetes.

Our study has certain limitations, including the relatively small cohort, which is further limited by incomplete data for some participants, including their comorbidities, such as NAFLD, which may have independent effects on BCAA levels and their associations with IR markers. Our study also had a short follow-up duration of 12 months. Further, given the observational nature of our study, the associations observed in our study do not prove causal relationships between BCAAs and IR. Additional studies with larger sample sizes and longer follow-up durations are essential to further characterize these relationships. Our study also has several strengths, particularly the inclusion of a non-surgical control group. This study, to our knowledge, is the first to report on changes in BCAA levels after SG, and their correlations with IR and BCF markers in adolescents and young adults.

## 5. Conclusions

In conclusion, we demonstrate a reduction in serum BCAA levels following SG in youth. Baseline BCAA concentrations are positively associated with weight and measures of IR, and the reduction in serum BCAA following SG is associated with a reduction in the measures of IR. Future studies are required to understand the causal relationship between BCAA and IR, and whether interventions that aim to reduce serum BCAA levels can be implemented in the treatment of IR and type 2 diabetes.

## Figures and Tables

**Table 1:** Baseline characteristics and changes over 12 months in clinical characteristics, IR and BCF markers, dietary intake, and serum levels of BCAA for sleeve gastrectomy and non-surgical groups.

	Baseline Measure		Changes over 12 Months		*p*-Value Comparing Changes over 12 Months in Surgical vs. Non-Surgical Groups
	Surgical (*n* = 30)	Non-Surgical (*n* = 32)	Surgical (*n* = 30)	Non-surgical (*n* = 32)	
**Clinical Characteristics**					
Age (years)	18.21 ± 0.38	17.83 ± 0.52	-	-	-
Sex (Female/Male)	23/7	24/8	-	-	-
Race (White/Black/Other)	18/7/5	17/6/9	-	-	-
Weight (kg)	133.4 ± 4.6 *	119.7 ± 3.8	−35.0 ± 2.5 ^¶^	0.6 ± 1.5	**<0.0001**
BMI (kg/m^2^)	45.11 (42.16, 52.05) *	41.65 (37.97, 46.85)	−12.02 (−15.31, −8.72) ^¶^	0.26 (−1.24, 1.64)	**<0.0001**
BMI z-score	2.50 (2.34, 2.77)	2.43 (2.23, 2.68)	−0.69 (−0.98, −0.42) ^¶^	−0.03 (−0.10, −0.002) ^¶^	**<0.0001**
**Dietary Intake (g)**					
Isoleucine	4.06 ± 0.34	3.67 ± 0.37	−0.92 ± 0.38 ^¶^	−0.16 ± 0.50	0.24
Leucine	6.72 ± 0.56	6.16 ± 0.63	−1.37 ± 0.66 ^¶^	−0.19 ± 0.80	0.26
Valine	4.40 ± 0.35	4.07 ± 0.42	−0.98 ± 0.41 ^¶^	−0.17 ± 0.53	0.24
BCAAs	15.18 ± 1.25	13.91 ± 1.42	−3.28 ± 1.44 ^¶^	−0.52 ± 1.82	0.25
**Serum Levels (uM/L)**	***n*** **= 21**	***n*** **= 23**	***n*** **= 20**	***n*** **= 19**	
Isoleucine	103.8 ± 7.1	97.7 ± 3.8	−23.8 ± 6.5 ^¶^	−4.8 ± 4.0	**0.02**
Leucine	185.4 ± 10.5	172.8 ± 6.1	−40.9 ± 7.6 ^¶^	1.7 ± 6.2	**0.0001**
Valine	161.1 ± 7.5	146.7 ± 6.0	−43.3 ± 6.6 ^¶^	−0.2 ± 4.4	**<0.0001**
BCAAs	450.3 ± 24.0	417.2 ± 14.4	−253.3 ± 21.8 ^¶^	−179.9 ± 9.2 ^¶^	**0.0004**
**IR and BCF Markers**					
HOMA-IR	2.49 (1.81, 4.15) (*n* = 28)	2.35 (0.99, 4.09) (*n* = 30)	−1.50 (−3.11, −1.13) ^¶^ (*n* = 27)	0.37 (−0.58, 1.82) (*n* = 29)	**<0.0001**
HbA1c, %	5.50 (5.30, 5.70) (*n* = 30)	5.44 (5.20, 5.77) (*n* = 32)	−0.40 (−0.60, −0.19) ^¶^ (*n* = 29)	−0.10 (−0.30, −0.02) ^¶^ (*n* = 31)	**0.002**
Insulinogenic Index	4.21 (2.48, 6.12) (*n* = 23)	4.08 (2.41, 6.03) (*n* = 29)	−0.42 (−1.94, 1.63) (*n* = 22)	0.57 (−0.16, 2.54) (*n* = 26)	0.10
Matsuda Index	3.87 (1.88, 5.88) (*n* = 22)	3.05 (2.06, 7.59) (*n* = 29)	4.36 (1.61, 8.40) ^¶^ (*n* = 18)	−0.96 (−4.02, 0.32) ^¶^ (*n* = 25)	**<0.0001**
Oral Disposition Index	0.25 (0.16, 0.40) (*n* = 23)	0.28 (0.19, 0.66) (*n* = 29)	0.40 (0.11, 0.80) ^¶^ (*n* = 22)	0.01 (−0.35, 0.42) (*n* = 26)	**0.02**

* *p* < 0.05 for between group comparisons at baseline. ^¶^ *p* < 0.05 for within group significance for changes over 12 months. Abbreviations: BCAAs: branched-chain amino acids; BMI: body mass index; HbA1c: glycated hemoglobin; HOMA-IR: Homeostatic Model Assessment for Insulin Resistance. Means ± SEM or Median (first quartile, third quartile) are reported. Significant *p*-values are in bold. For comparisons across groups for baseline measures and differences over 12 months, we used Student’s *t*-test for parametric data and the two sample Wilcoxon rank sum test for non-parametric data. Within group changes over 12 months were assessed using the paired *t*-test or the Wilcoxon sign rank test.

## Data Availability

The data are not publicly available as this study is still ongoing.
